# Multi-Agent Credit Assignment and Bankruptcy Game for Improving Resource Allocation in Smart Cities

**DOI:** 10.3390/s23041804

**Published:** 2023-02-06

**Authors:** Hossein Yarahmadi, Mohammad Ebrahim Shiri, Moharram Challenger, Hamidreza Navidi, Arash Sharifi

**Affiliations:** 1 Department of Computer Engineering, Science and Research Branch, Islamic Azad University, Tehran 1477893855, Iran; 2 Department of Computer Science, University of Antwerp, 2020 Antwerp, Belgium; 3 Flanders Make Strategic Research Center, 3001 Leuven, Belgium; 4 Department of Mathematics and Computer Science, Shahed University, Tehran 3319118651, Iran

**Keywords:** smart cities, resource allocation, multi-agent system, credit assignment problem, bankruptcy game, reinforcement learning

## Abstract

In recent years, the development of smart cities has accelerated. There are several issues to handle in smart cities, one of the most important of which is efficient resource allocation. For the modeling of smart cities, multi-agent systems (MASs) can be used. In this paper, an efficient approach is proposed for resource allocation in smart cities based on the multi-agent credit assignment problem (MCA) and bankruptcy game. To this end, the resource allocation problem is mapped to MCA and the bankruptcy game. To solve this problem, first, a task start threshold (TST) constraint is introduced. The MCA turns into a bankruptcy problem upon introducing such a constraint. Therefore, based on the concept of bankruptcy, three methods of TS-Only, TS + MAS, and TS + ExAg are presented to solve the MCA. In addition, this work introduces a multi-score problem (MSP) in which a different reward is offered for solving each part of the problem, and we used it in our experiments to examine the proposed methods. The proposed approach is evaluated based on the learning rate, confidence, expertness, efficiency, certainty, and correctness parameters. The results reveal the better performance of the proposed approach compared to the existing methods in five parameters.

## 1. Introduction

Smart cities have rapidly developed in recent years. To develop these systems, some issues should be considered [1]. One of the most important of these issues is resource allocation [2]. There are many resources that should be assigned and used in smart cities, including smart transportation resources [3], smart healthcare resources [4], smart home resources [5], smart energy resources [6], smart factory resources [7], and so on. Multi-agent systems (MASs) are suitable tools for modeling smart, intelligent, and distributed systems [8]. In this sense, smart cities can be mapped and modeled into MASs. To propose a method for resource assignment in smart cities, we use multi-agent reinforcement learning (MARL) and the multi-agent credit assignment (MCA) problem.

In MARL, agents interact with the environment, which results in rewards/punishments. The environment delivers their resultant to the MAS as a global reward [9]. Now, this global reward must be distributed among the agents to help in the learning of each agent. In other words, MARL agents are taught via the reactions from the environment and by receiving the reward/punishment. The environment returns the resultant of these rewards/punishments as global rewards to the critic, and the critic should find a method to distribute these among the agents. This process continues until the learning among the agents reaches an acceptable level. In the mapping of smart cities’ resource allocation into MASs, every resource, including roads, bandwidths, processors, etc., can be mapped into the global reward and every resource consumer mapped into the agent. Here, we are faced with the essential question of how this global reward (resource) is going to be distributed among the agents (consumers) to have better learning and lead to more efficient resource allocation. In the relevant literature, this is known as a multi-agent credit assignment problem (MCA) [10,11]. We can consider resource allocation as an MCA problem in which the resource as a global reward should be distributed among the consumers as agents. There are many methods to solve the MCA, as there are many methods to solve the resource allocation problem. One of the important goals of solving the MCA is increasing the efficiency, to which less attention has been paid in the literature. In other words, the goal of many resource allocation methods is only the allocation of resources between the consumers, and the efficiency of the systems is not the focus point.

Basically, there are two approaches to solving the MCA problem. In the first approach, called the fair approach, authors have mainly sought to determine the true share for each agent from the global reward [11,12]. In the second approach, which we call the efficiency approach, we seek to assign credit among the agents so that the efficiency of the MAS increases. The latter approach has received less attention in related papers. In this work, we tried to use the second approach, which was to increase the efficiency of the MAS. If we map the smart cities into the MAS, then the resource allocation in smart cities can be examined from these approaches. This has been performed in most related works in this area, as the goal of the authors was resources to all devices as consumers. However, the efficiency of these systems such as smart cities has been less investigated. Therefore, in order to increase their efficiency, we attempt to solve the resource allocation in smart cities in the second approach and viewpoint. In our work, we attempt to apply the proposed method to increase efficiency, so these methods may be in conflict with the fair approach.

In most of the studies conducted in this area of research, the operation environments were considered uniform. This means that solving any section of the problem results in the same rewards or punishments as other sections [13]. This is despite the fact that there are several problems, which are non-uniform and multi-score in nature, for which solving their different components will lead to different rewards or fines. Although there are many studies on uniform problems [10,13], these types of problems have received less attention than uniform problems. In this paper, our focus is on these types of problems, and we present a puzzle problem as an operation environment, which we call the multi-score puzzle (MsP). In most papers related to this subject, the agent begins to perform their task from the zero point [14]. It should be noted that there are many problems in which agents only begin doing their job if they receive a suitable reward; otherwise, they do nothing. These kinds of issues are often seen in everyday human life, such as the problem of workers [15], in which workers begin to do their work only if they receive adequate wages; otherwise, they do not work. Therefore, it seems necessary to consider these kinds of issues. This is why we introduced a new constraint and named it the task start threshold (TST). This constraint means that each agent will start to work and participate in the MAS only if it receives a reward at least equal to this TST; otherwise, it will not start to work.

In this paper, considering this constraint and global reward, we turn the MCA into a bankruptcy game and introduce a new method according to the bankruptcy game to solve it. The problem of bankruptcy deals with how to divide an inadequate resource among the agents. This method was applied to financial and economic as well as engineering fields. It is a subfield of game theory, first presented by O’Neill [16]. In this paper, the global reward considers the property of a debtor (a limited resource), which should be distributed among the agents as creditors. This means that we are faced with a bankruptcy problem. Introducing a new multi-score problem as an operating environment called MSP is the first contribution to this study. In the second, a new constraint called TST was introduced to deal with the problem that agents begin to do their tasks upon receiving a suitable reward, and a final important contribution was that of proposing a basic method according to the bankruptcy game for solving the MCA.

The rest paper is organized as follows: In Section 2, we will review the related work. The required preliminaries and definitions are introduced in Section 3. The proposed bankruptcy-based method for solving the MCA problem and its related methodology are presented in Section 4. The results of the simulation of the proposed methods and a comparison of them with the existing methods can be found in Section 5. In Section 6, we will discuss proposed methods. Finally, Section 7 will provide conclusions as well as a technical outlook.

## 2. Related Work

It is anticipated that in 2050 more than half of the world’s population will dwell in urban regions [17]. Smart cities involve many smart objectives, such as smart transportation, smart healthcare, smart homes, etc. [18]. In all of these smart objectives, resource allocation is an essential problem [19]. Many subjects, such as roads in transportation, bandwidth in smart homes, and access to doctors and medicine in healthcare, can be considered resources [20]. These resources should be allocated to the people and to the consumers. Smart cities can be considered MASs in such a way that every consumer is mapped as an agent [21]. In addition, for efficient resource allocation in smart cities, MARL and MCA can be used. RL was based on animal psychology research [22], where learning was based on reward and punishment. In RL, learning happens through repetition as well as a trial and error process that makes it a powerful approach to dynamic and unknown environments [23]. Due to the features of RL, this method was developed for distributed systems, one of which is multi-agent environments [24]. In MARL, agents interact with the environment and then, based on their actions, receive a global reward from the environment, which must be distributed among the agents [10,11,13,25]. Thus, one of the most important issues of MARL is the MCA, which means the distribution of reward/punishment among the agents. MCA was first introduced by Skinner in experimental psychology [26]. Skinner introduced the MCA in such a way that the success of a system depends on the cooperation of its constituents and a particular presentation of the reward/punishment sharing problem. MCA can be taken into account from two aspects, i.e., an equal credit assignment approach [27] and a fair credit assignment approach [28]. In the first approach, the global reward is distributed equally among the agents [29,30], while in the second approach, the rewards are distributed between the agents based on their participation levels. For the second approach, the fundamental question is how this participation is defined. In order to answer this question, methods such as differential rewards [31] and the Shapley method [32] have been proposed. In the classification of the credit assignment problem (CA) by Rahaie and Beigy [13], the CA has been classified into four categories which include:

1. Temporal CA
2. Structural CA
3. Social CA
4. Multi-agent CA

In the temporal CA, the agent interacts with the environment, but the action’s result does not deliver to it immediately; therefore, when an agent receives feedback and a reward from the environment, they do not know exactly which action was relevant to the reward and which action merited the reward. Three models of reinforcement learning in this aspect include [33]:

- Actor–critic
- Q-learning
- SARSA

If we consider the agent as an entity that has the knowledge and acts based on that knowledge, then we will be faced with the same question as in the structural CA [34], and we must answer the part of the knowledge which is worthy of the reward. In Social CA [35], the CA has been considered from a social point of view. In [35], a computational model based on the attribution theory of psychology has been proposed. The last type of CA that we faced within this study was the MCA, which is considered a part of the MARL’s process. In this paper, we will focus on the MCA in which agents act independently. One of the first works in MCA was the knowledge-based reward assignment [10] wherein the reward is distributed among the knowledge-based agents. Each agent’s knowledge is assessed by certain criteria and then sorted. In [13], two methods have been proposed for solving MCA, the first being based on the history of the agent’s interactions and the second based on its knowledge. These methods were developed in [10]. In the history-based method for solving the MCA, previous knowledge was used for assigning credit to the agents of MASs. In this method, an undirected graph is used for modeling the environment. First, a set of variables without a value is introduced for environmental modeling, and the environment model will be completed as soon as the values are defined. This method uses the history of the agents in the distribution of the rewards, but it requires constructing a model based on a graph of the interactions between the agents and the environment. If we consider the MARL as divided into two categories, i.e., model-based and model-free [36], then we can say that this method is model-based in which building a model takes time. The issue becomes more complicated when the number of graph nodes increases, which results in difficulties in model processing. The second method for solving the MCA proposed in [13] is referred to as the ranking method. Based on the agent’s knowledge assessment, the ranking-based method requires metrics to measure that knowledge. After these criteria were determined, the next step is to sort the agents according to those metric(s). The final step of this method is to distribute the rewards according to the sorted list. In this method, it is possible that the whole global reward is assigned to the agent with the highest rank, and no reward is assigned to any other agent. Therefore, it may lead to the result that the MAS becomes a single-agent system. The next method for solving the MCA problem is called the dynamic method [37]. In this method, the global reward is dismembered into weighted reward summation between the agents. In this method, the share of each agent in the global reward is determined based on one of the criteria presented in [37]. Although it is attempted to use one of the criteria for the agent’s knowledge, this reward will be merely based on one parameter and may not be accurate enough in assigning the reward to the agents. Therefore, this method may not only lead to the unfair distribution of rewards among the agents but also may give rise to a decrease in the efficiency of the MAS. An MAS can be considered a cooperative game between agents [38]. Unlike competing with others [39], agents in cooperative games aim to solve a common task or maximizing the overall payoff to collaborate with each other. Therefore, from this perspective, the MCA problem can also be considered a profit-sharing problem in cooperative games [40], for which various methods such as the Shapley method [41], the core [42,43], Nash bargaining [43], and bankruptcy [44,45] have been suggested for. One of the methods used to allocate resources when they are limited and there are many applicants is the bankruptcy method [45]; this happens when the available resources are less than what is requested by the applicants. Since we are facing a global reward issue, which is a limited resource and ought to be appropriately distributed among the agents applying for the reward, we are facing bankruptcy. We also used this method (bankruptcy) in the present paper. Table 1 summarizes the methods to solve the MCA problem in the literature and compares them with the approaches suggested in the paper. This table compares these methods based on the approach type, their contributions, advantages, and disadvantages, as well as specifies the conditions under which any method is suitable.

## 3. Preliminaries

### 3.1. Definitions

This section describes the notation employed in the present paper. The any agents with *Ag* and *i*th agent are represented with ${Ag}_{i}$. Assume that the MAS is composed of *N* agents, then we have $MAS=\{{Ag}_{1},{Ag}_{2},\ldots ,{Ag}_{N}\}$. According to the TST constraint, if the received reward of an agent is more than a particular value, the agent begins doing its tasks. As a result, the reward obtained by agent *i* at time *t* will be presented with $r_{i}^{t}$, while ${TST}_{i}$ indicates its TST. In accordance with the TST, any agents can be inactive or active. As a result, at time *t*, each active MAS and active agent are shown by ${MAS}^{t}$ and ${Ag}_{i}^{t}$, respectively, in such a way that ${MAS}^{t}=\left\{{Ag}_{1}^{t},{Ag}_{2}^{t},\ldots {Ag}_{n}^{t}\right\}$. At time *t*, an action is carried out by every active agent ${Ag}_{i}^{t}$ in the environment that is shown by $a_{i}^{t}$. At time *t*, $A^{t}$ indicates the actions set pertaining to the agents within the environment. As a result, we can write $A^{t}=\left\{a_{1}^{t},a_{2}^{t},\ldots ,a_{n}^{t}\right\}$. Given that the MAS is considered by the environment as a single entity, for the action of each agent, the resultant punishments/rewards are returned as a global reward to the entity known as the “critic”. One can represent such a global reward as *R*. Given that such a global reward does not remain the same constantly at time *t,* we can display it by $R^{t}$. The critic is responsible for the distribution of the received global reward among the agents. To put it another way, it is the task of the critic to create the vector $\left(r_{1}^{t},r_{2}^{t},\ldots ,r_{n}^{t}\right)$ in such a manner that $\sum_{i=1}^{n}r_{i}^{t}=R^{t\;}$. In the present paper, the agents’ knowledge on the basis of the parameters presented in [10,13] is employed. Therefore, in order to arrange the agents in accordance with their associated knowledge, the symbol *“≪”* is employed; thus, in order to show that agent *j*’s knowledge is greater than agent *k*’s knowledge, we write ${Ag}_{k}\ll {Ag}_{j}.$ Furthermore, the function $SAg\left(.\right)$ on the basis of the TST constraint shows that the agent begins working in accordance with the conditions in Equation (1):

(1) $$SAg\left(r\right)=\left\{\;\begin{array}{l} \mathrm{True}\;\;\;\;\;\;\;\;\;\;\;\;\;\;,\;\;r\geq \;TST\\ \mathrm{False}\;\;\;\;\;\;\;\;\;\;\;\;\;\;,\;\;r<TST \end{array}\right.$$

In the present paper, the *Q* learning technique is utilized for the RL of the agent [46]. Furthermore, each agent must resolve part(s) of the problem. As soon as some parts of the problem are solved by ${Ag}_{i}$ at time *t*, the amount of the global reward provided by the environment is decreased/increased by $c_{i}$. In this paper, such a global reward decrease/increase is denoted by $R_{r_{i}^{t}}^{t}↑c_{i}$ */* $R_{p_{i}^{t}}^{t}↓c_{i}$. If $Sc=\left\{i:\;{Ag}_{i}\;\mathrm{is}\;\mathrm{successful}\;\mathrm{at}\;\mathrm{time}\;t\right\}\;$shows the set of successful agents, then:

(2) $$r_{i}^{t_{\mathrm{new}}}=r_{i}^{t_{\mathrm{old}}}+c_{i\;}\;$$

(3) $$R^{t_{\mathrm{new}}}=\;R^{t_{\mathrm{old}}}+C\;,\;where\;C=\sum_{i\in Sc}^{}c_{i}\;\;\;\;\;\;\;\;$$

### 3.2. Bankruptcy Concepts

In order to assign credits to agents, the present paper employed the concept of bankruptcy. The problem of bankruptcy is a part of the cooperative game theory, and it attempts to define the method of distributing a debtor’s assets among their creditors when those assets are not enough to meet their total claims; thus, the amounts allocated to every single creditor is a non-negative value, which is not higher than the amount claimed [45].

#### 3.2.1. Definition 1: Bankruptcy Problem

If the claim raised by each creditor is denoted by $d_{i}$, the debtor’s assets are shown by *E*, $D(d_{1},d_{2},\ldots ,d_{N})$ stands for the vector of the creditors’ claims, *ReqD* indicates the sum of creditors’ claims, $x_{i}$ represents the part of the debtor’s assets dedicated to every single creditor, and *N* shows the number of debtors, we can write:

(4) $$E=\sum_{i=1}^{N}x_{i}\leq ReqD=\sum_{i=1}^{N}d_{i}\;\;\;\;\;\;$$

(5) $$0\leq x_{i}\leq d_{i}\;\;\;\;\;$$

Equations (4) and (5) are called feasible equity and reasonable conditions, respectively.

#### 3.2.2. Definition 2: Bankruptcy Game

If the pair $\left(E,D\right)$ is regarded as a bankruptcy problem, then an *n*-fold assignment constitutes a solution to the problem of bankruptcy $\left(E,D\right)$, where $E=\sum_{j\in N}^{}x_{j\;}$. To select the mode leading to maximum efficiency, one can use an allocation function. In the course of the development of the allocation function in a cooperative game characterized by *n* players, one can define the pair (N,c) in such a manner that N={1,2,…,n} is representative of a finite set of players, $\;2^{n}$ stands for the number of subsets of *N*, the allocation function is shown by $c:2^{n}\to R$, and $c\left(⌀\right)$ is considered to be 0. As a matter of fact, we consider the S subsets of N as allocations in such problems, and the amount of c(S) is presented as the *S* value (asset). In the course of the allocation, the asset of every single player will be interpreted as its maximum cost/profit. Here, assume a constant set of players by a game (N,c) in which *c* stands for an allocation function. Then, one can define the bankruptcy game in accordance with the bankruptcy problem (E,D) using Equation (6),

(6) $$c_{E,d}\left(S\right)=max\{(E-\sum_{j\in N\backslash S}^{}d_{j})\;,0\}\;\;\;\;\;\;$$

To solve the bankruptcy problem, many methods have been proposed. In this paper, we use the AP rule, which is one of the classic methods.

#### 3.2.3. Adjusted Proportional Bankruptcy Rule (AP Rule)

In accordance with this technique [47], as a primary allocation to the person *i*, all the claimants’ needs except for those of person *i* are first satisfied via Equation (7).

(7) $$v_{i}=\mathrm{Max}\left\{0,E-\sum_{j\neq i}^{}d_{j}\right\}\;\;\;$$

The remainder is subsequently dedicated to claimant *i*, and if a negative value is determined as the remainder, or nothing is left, a value of zero is dedicated to the same claimant. Such a process is explained by Equation (8).

(8) $$x_{j}=v_{i}+\left(d_{j}-v_{i}\right){\left(\sum_{j\in N}^{}(d_{j}-v_{i})\right)}^{-1}\left(E-\sum_{j\in N}^{}v_{j}\right)\;$$

In Equations (7) and (8), $\;v_{i}$ stands for the share of claimant *i*. As soon as $v_{i}$ is calculated, one can calculate the share of every single claimant, namely *j*, on the basis of Equation (7), in which *N* stands for the claimants’ number. Given that in this technique, other representatives are initially taken into account and prioritized in order to decide the initial allocation of claimant *i*, one can call the initial allocation the minimum claimant *i.*

### 3.3. Problem Definition

On the basis of effectiveness and fairness, one can characterize the MCA problem as:

1. Fair–inefficient;
2. Fair–efficient;
3. Unfair–inefficient;
4. Unfair–efficient.

The ideal condition can be regarded as a state in which the efficiency is increased besides being characterized by a fair distribution. The investigations carried out in the same field have tried to continue on the same path; however, fair distribution has often been scrutinized. There are currently numerous problems that lead to efficiency reduction instead of increasing efficiency. As a result, the MCA has two more general cases:

- Unfair and efficient;
- Fair and inefficient.

**Figure 1:**
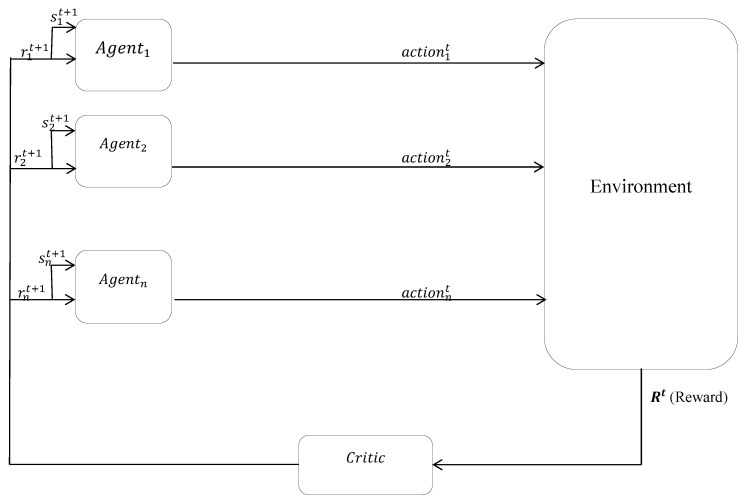
MCA process.

The present investigation attempts to enhance the MAS efficiency in such a way that all agents take advantage of this increased efficiency. If the reward received by the agents from the environment via the critic is less in comparison with their TST values, the problem takes on a more challenging aspect. In such cases, a number of agents will not begin their work. To put it another way, the amount of received reward obtained from the environment via the critic is lower in comparison with the agents’ TSTs, and as a result, we are faced with the bankruptcy problem. Figure 1 indicates the interaction between the agents and the environment. As shown in Figure 1, there is an MAS that consists of some agents which perform actions in the environment. The environment, based on the correct/incorrect action of any agent, generates a partial reward/punishment for any agent’s action. The environment returns the resultant of these partial rewards/punishments in the form of a global reward to the critic (it is shown by $R^{t}$ (reward)). At this point, the critic encounters the question of how the global reward should be distributed among the agents. We attempt to answer this question in this paper.

In other words, it can be said that a task is assigned to every single agent that attempts to conduct it. Such a task may be carried out incorrectly/correctly. As soon as this interaction is accomplished, a global reward is returned by the environment as the reward resulting from the actions of the agents to the MAS, which is then received by the critic. At this moment, the critic encounters the problem of distributing the global reward, which is lower in comparison with the sum of TSTs amongst the agents (despite being potentially unfair), leading to the increased efficiency of the MAS. The present paper seeks to answer this question and present some solutions to the same problem.

### 3.4. Multi-Score Puzzle

**Figure 2:**
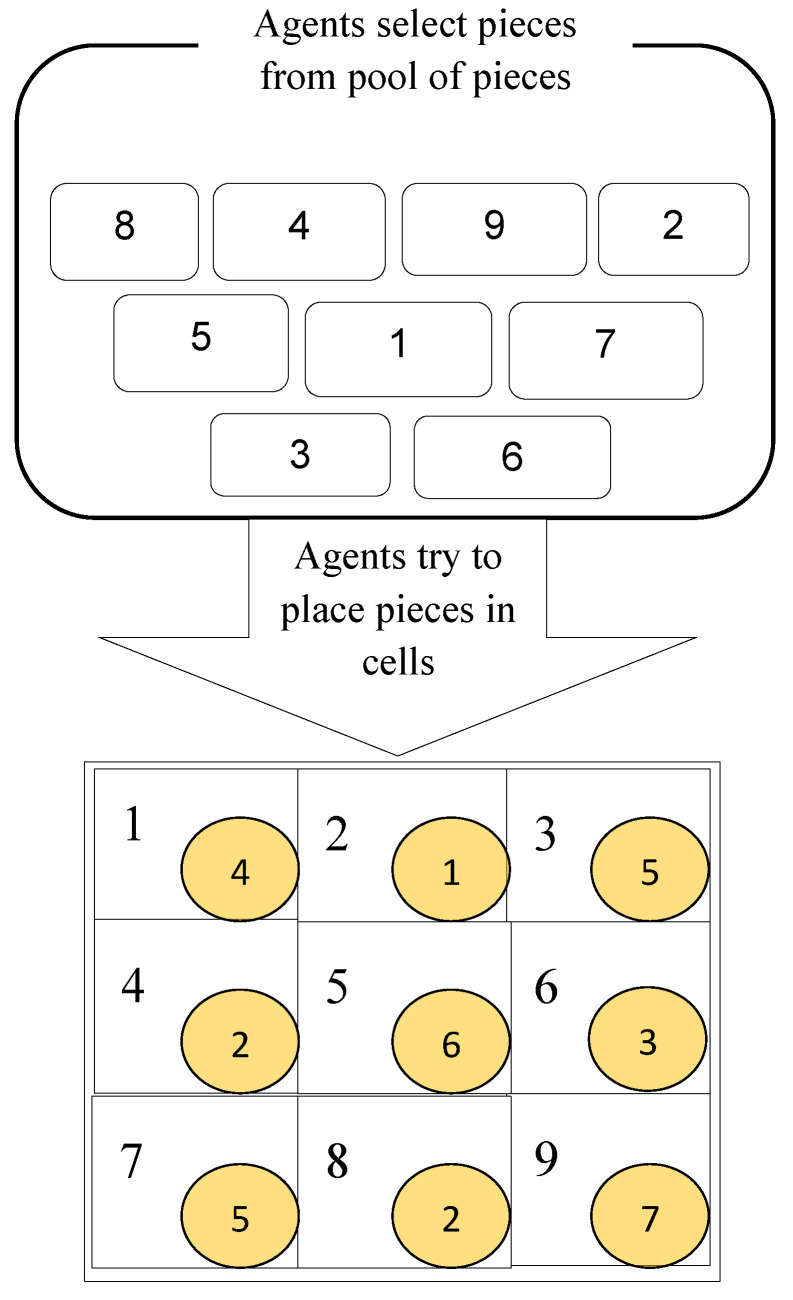
Multi-score puzzle scheme.

The environment shown in Figure 2 is an MSP. The MSP introduced in this paper is a multi-score puzzle (MsP) that includes some cells and pieces, and any agent is responsible for filling some of these cells with appropriate pieces. The most important issue about this puzzle is that filling any cell, with right or wrong pieces, has a different point compared to other cells; for this very reason, we called it MsP.

Figure 2 illustrates an MsP problem. The scores are for filling a cell with the correct pieces in a small square located at the bottom of that cell. It should be noted that the punishment for filling a cell with a wrong piece is zero in our work. There is a pool of pieces in this figure that from which any agent can select several pieces and, using their knowledge, put the selected pieces in a suitable cell. Any agent is responsible for solving a section of the puzzle that contains some cells.

## 4. Methodology

### 4.1. Reverse Adjusted Proportional Algorithm (RevAP)

In this section, taking the TST constraint into account in order to solve the MCA problem as a bankruptcy problem, first, a basic method based on bankruptcy is introduced. Then, three new methods are presented on the basis of this basic method. The operating environment was an MsP, as described in Section 3.4. In this environment, an MAS is used to solve the puzzle. Each agent has the responsibility of placing one or more pieces in the correct location. The presented fundamental technique was modified on the basis of the adjusted proportional rule (AP); however, it operates in reverse, and hence it is named reverse AP. In addition, the presented technique employed the ranking concept so as to rank and prioritize the agents on the basis of their knowledge. Based on the AP technique, except for claimant *i*, the whole claimants’ demands are initially met; subsequently, if anything remains from the debtor’s assets, the claimant *i*’s demands will be met. In the case that the remained reward is negative/zero, nothing will be allocated to the claimant *i*. In the same technique, the agent characterized by the minimum request is the claimant *i*. In contrast, in the proposed method, first, the request by claimant *i* is satisfied and then the request by the other claimant is processed. In the proposed method, the claimant *i* is the most knowledgeable agent among the agents to which no reward has yet been awarded. We ranked the agents in accordance with their knowledge to prioritize them. As a result, according to the agents’ knowledge, they were sorted in descending order with regard to the definition of the agents’ arrangement; subsequently, the allocation of rewards was carried out. Such prioritization is shown in Equation (9):

$${Ag}_{k}\gg {Ag}_{s}\gg \ldots \gg {Ag}_{p},$$

(9) $$\forall {Ag}_{i}\in MAS\;,\;i=\left\{1,2,\ldots N\right\}\;\;$$

We employed the reverse AP technique in order to assign the rewards to agents. This means that the rewards were initially assigned to the agent characterized by the maximum level of knowledge, and this allocation process followed a descending order on the basis of their knowledge. To launch the process, the minimum reward given to an agent must be on par with its TST. As a result, if it is possible, the critic must assign the minimum possible amount of reward, namely, that equal to TST, to the agents in accordance with the previously described technique. When the mentioned technique is employed to reward the agent characterized by the maximum knowledge, the agent characterized by the second-best knowledge must be rewarded in descending order. The same process is continued until no reward remains. Equation (10) depicts the method used to allocate the rewards to every single agent, while Equation (11) depicts the updating process of the available rewards.

(10) $$r_{i}^{t+1}=\left\{\begin{array}{l} {TST}_{i},\;\;\;\;\;\;\;\;R^{t}-{TST}_{i}\geq \;0\\ \;\;0\;,\;\;\;\;\;\;\;\;\;\;\;\;\;\;\;\;\;\;\;\;\;o.w \end{array}\right.$$

(11) $$R^{t}=R^{t}-{TST}_{i}\;\;\;\;\;\;\;$$

In Equations (10) and (11), $TST_{i}$ stands for the $Ag_{i}$’s TST, $r_{i}^{t+1}$ indicates the credit assigned to $Ag_{i}$ at time *t* + 1, and $R^{t}$ shows the global reward. If T.i stands for the order of the agents between the agents on the basis of their knowledge, then ${Ag}_{T.N}$ and ${Ag}_{T.1}$ have the least and the most knowledge, respectively, then the “Reverse Adjusted Proportional” *(RevAP)* algorithm represents the mentioned Algorithm 1.
 **Algorithm 1:** Algorithm RevAP  Inputs: Vector of Agents’ Reward **r**(**r**_T.1_, **r**_T.2_, …, **r**_T.n_) where for any **r**_T.i_ = 0  R: Global Reward  Outputs: Valued Vector **r**(**r**_T.1_, **r**_T.2_, …, **r**_T.n_)1.    Agents were arranged according to their TSTs2.    i = 13.        Repeat4.             if R ≥ TST_T.i_5.                 Begin6.                      **r**_T.i_ = TST_T.i_7.                      R = R − **r**_T.i_8.                 End9.        i = i + 110.   Until ((R ≤ 0) OR (The residual reward is not sufficient to assign any agent))

In the case of a positive residual reward after the process completion and allocation of the rewards, which is not allocable to the agents in accordance with Equation (10) (namely, insufficient to launch an agent), one must distribute the residual reward among those agents to which the reward has already been allocated. Here, this question should be answered: “how should one distribute the above residual reward among the agents?” Three different techniques are presented below to answer the above question.

- TS-only method.

This technique initially assigns the credit to the agents in accordance with the *“RevAP”* algorithm. If the residual reward is positive but not allocable to other agents (the residual value is lower compared to the TST value of the subsequent agents on the basis of their knowledge order), such a reward will be ignored. To put it another way, this technique simply attempts to make the agents begin working with the minimum amount of rewards.

- TS + MAS priority method (TS + MAS method).

This technique serves on the basis of the TS-only method. The above two techniques are nearly the same, except for the fact that the residual reward, denoted by $R_{rem}$, will not be ignored in the latter if the residual remains positive after being allocated. In the same technique, such a residual value is allocated based on the requirements of every single active agent, as in Equation (12),

(12) $$r_{i}^{t}=r_{i}^{t}+\left(\frac{{\left(r_{i}^{t}\right)}^{2}}{\sum_{i=1}^{n}{\left(r_{i}^{t}\right)}^{2}}\right)\times R_{\mathrm{rem}}$$

- TS + expert agent priority method (TS + ExAg method).

This technique serves in much the same way as the TST + MAS technique; however, the residual reward is distributed differently. This technique assigns the remaining reward to the most knowledgeable agent of $MAS^{t}$. If such an agent is denoted by $Ag_{knowledeable}$, this residual value will be allocated in accordance with Equation (13),

(13) $$r_{i}^{t}=\left\{\begin{array}{l} r_{i}^{t}+R_{\mathrm{rem}},\;\;i=knowledgeable\\ \;\;r_{i}^{t\;}\;\;\;\;\;\;\;\;\;\;\;\;\;\;\;\;\;\;\;,\;\;\;o.w \end{array}\right.$$

### 4.2. MARL as Execution Platform

MARL is a multi-section process, and one of its sections is MCA. MARL consists of two phases, including the training phase and the test phase.

#### 4.2.1. Training Phase

Due to the agents having no knowledge at the beginning of the training phase, they begin working through a random selection of the piece(s) that should be situated in the right place. Subsequently, they choose the cells wherein to place such pieces. These selections may be false or true. When the whole pieces are situated within the cells (some in the wrong and some in the correct cells), a global reward is returned by the environment on the basis of the way in which the pieces are placed within the cells, which results from the punishments and rewards. Here, we must answer the fundamental question of the present work “how should the global reward be distributed among the agents by the critic?” Given that the critic does not have any information regarding the agents’ knowledge, they distribute the global reward ($R^{t}$) equally amongst the agents in this phase. To put it another way, Equation (14) is used to determine the share of every single agent from the global reward,

(14) $$r_{i}^{t+1}=\frac{R^{t}}{N\;}$$

In Equation (14), the agents’ number indicated by *N*, the global reward that the critic received from the environment and should distribute among the agents, was shown by $R^{t}$. The agents’ learning occurred based on the RL method. In this study, we used the Q-learning method for agent learning; therefore, each agent updated its Q-table with the reward received from the critic. At the end of the training phase, the agents will be ranked by their knowledge that the critic has evaluated based on the criteria defined in [13]. In other words, at the end of the training phase, the critic can evaluate the agent’s knowledge based on the Q-table of any agent. This evaluation is based on the criteria that are defined in [13], which can be extracted from the agent’s Q-table. After that and at the end of the training phase, the critic can rank the agents based on their knowledge. In the next phase, the test phase, the critic applies the ranking of agents in order to assign the reward to agents based on the proposed methods.

It should be noted that because the agents are trained in the training phase, the learning will occur during these, as mentioned in [10,13]. Those agents that were more successful in learning will perform better. This better learning will cause the agents to make a better choice in the test phase and select pieces and cells with higher scores. In other words, at the end of this phase, agents will be able to find suitable pieces and cells based on their learning and the training they receive. One more issue to consider in this phase is how to find and configure the TST values for the agents. As stated before, global rewards are distributed among the agents according to their TST values. Therefore, we need a way to find and adjust it. Various methods may be considered for this, among which there are trial-and-error methods, evolutionary methods, and RL [48]. Since there is not much information regarding the agents in this phase and given the fact that the agents are located in an unknown environment, RL appears to be an appropriate method to do this. In this work, we used RL to find the TST values in this phase. Algorithm 2 shows the training phase.
 **Algorithm 2:** Algorithm Training.  Inputs: MAS  Outputs: A ranked list of agents based on their knowledge1.    Repeat2.          Ag_i_ ∈ MAS^t^ selects a subset of pieces from pieces pool3.          Ag_i_ ∈ MAS^t^ selects cells to fill it(them) with appropriate pieces4.          The environment returns a global reward to the critic (MAS)5.          The critic distributes the R among the agents equally6.          Any agent updates its Q-Table7.    Until training phase finishes8.    For any agent, calculates its knowledge via [10,13] and TST via RL method9.    Sort the agents according to their knowledge

#### 4.2.2. Test Phase

In this phase, the key question was how the reward should be allocated so that it solves the problem in addition to raising the agent’s knowledge. As stated earlier, if an appropriate reward assignment is not made, then each and every agent $Ag_{i}$ may never reach its $TST_{i}$, so its task will be delayed. The MCA problem can be considered as follows:

1. The reward that the critic receives from the environment and distributes among the agents is high enough so that all the agents start to work.
2. The reward that the critic receives from the environment and distributes among the agents is so low that no agent starts to work.
3. The reward that the critic receives from the environment is less than the total needs of the agents to reach their TST, but some of them can start to work anyway.

The first and second cases are not discussed in this paper. The problem we were interested in was the third case. For solving this problem, in other words, for the proper assignment by the critic, we used the bankruptcy concept, and three methods of TS-only, TS + MAS, and TS + ExAg were proposed. To implement the developed technique, one needs to prioritize the agents. As a result, the present paper gave priority to knowledgeable agents. From another perspective, in accordance with the RevAP employed in the present paper, the credits must be assigned to the agents. This means that the reward was initially allocated to those agents that had the highest priority, i.e., knowledgeable agents followed by reward distribution among other agents based on the order of their knowledge level. In the test phase, in each episode, any agent selects a piece of the puzzle and a cell to fill that cell with the right piece. Here, the knowledgeable agents, owing to their learning better than the other agents, act better than less knowledgeable ones in selecting the most valuable parts of the puzzle [10,13]. After each episode, in the test phase, the environment returns a global reward to the MAS, which is a resultant of the rewards/punishments for the agents’ actions. This global reward must now be distributed among agents so that it increases the MAS’ performance. To do this, the requests by more knowledgeable agents are initially met according to the proposed basic method. This means that, first, such agents will be given a reward at least equal to the TST value if possible. Consequently, these types of agents launched and try to solve the higher-scored section of the puzzle; therefore, the environment, according to Equations (2) and (3), gives a reward to $MAS^{t+1}$, which is greater than the reward given to $MAS^{t}$. This operation causes the reward allocated to the MAS to increase, and thus the critic can distribute more rewards among the agents in the new distribution. As a result, the agents that did not receive a reward other than their TST in the old time ($t^{old}$), will receive sufficient reward (at least equal to their TST) at the new time ($t^{new}$) and cooperate with other agents to solve the problem. In other words,

(15) $$\exists \;c_{k},{Ag}_{k}\in MAS\;\left(r_{k}^{t^{\mathrm{new}}}=r_{k}^{t^{\mathrm{old}}}+c_{k}\right)\geq {TST}_{k}$$

According to Equation (15), if an agent or agents, e.g., $Ag_{i}$, were to perform their task properly at time $t^{old}$, in accordance with Equations (2) and (3), the reward obtained by the MAS will increase by C. In other words, this reward increment by $Ag_{i}$ at time $t^{new}$, which is denoted by $c_{i}$ in Equation (2), will allow the critic to have more rewards than before (increase in amount C) and therefore be able to distribute more rewards among the agents. This higher value, considering the TSTs of the agents, can (may) lead to an increase in the reward of one or more agents (such as $Ag_{k}$ in Equation (15)) so that their received rewards will be greater than their TST, and therefore this agent or these agents will start to work and cooperate in problem-solving. This process will be repeated until the problem will be solved. Algorithm 3 illustrates the test phase.
 **Algorithm 3:** Algorithm Test.  **Inputs**:  MAS: a set of agents  Multi-score Puzzle: as the environment  **Outputs**:  Solved Puzzle  Score of game1.    *Initialize R (R^0^)*2.    *Initialize MAS ^0^*3.      ***Repeat***4.        *Reset Puzzle*5.            ***Repeat***6.                *Ag_i_*∈ *MAS ^t^ with r ^t^* ≥*TST_i_ selects pieces*7.                *Ag_i_*∈ *MAS ^t^ with r ^t^* ≥*TST_i_ select suitable places(cells)*8.                *The environment produces the global reward and delivers it to the critic*9.                *Based on the proposed methods the critic assigns the global reward into agents*10.              *The $MAS^{t}$ is updated*11.              *Any Ag_i_* ∈*MAS ^t^ updates its Q-Table*12.               ***if** Ag_i_ finishes its task then*13                   *MAS ^t+1^ = MAS ^t^ – {Ag_i_ : Ag_i_ finished its task}*14.              ***else***15.                   ***if** (SAg(r_i_^t^)==True)*16.                       *Goto 6*17.                   ***else***18.                      *Goto 8( Wait for the new distribution)*19.          ***Until** Puzzle Solved*20.   ***Until** Test Phase Finishes*

In the test algorithm, the agents are arranged based on their knowledge in the training phase, in function who received rewards which were higher than their TSTs, sob that they may first begin to select pieces of the puzzle. Then, according to their knowledge, they will start to select cells from the puzzle. It should be noted that during the training phase, the knowledge of the agents is evaluated based on [10,13]. The knowledge of the agents causes the more knowledgeable agents to be trained in a way that is capable of selecting those pieces and cells from the puzzle that have more scores with fewer yet more correct attempts. The greater their knowledge, the more accurate (and time-consuming) the selection of pieces and cells with more scores becomes. Conversely, the lesser their knowledge, the less accurate these selections become, and accordingly, taking longer time and making more mistakes in the choosing of pieces and cells. After the agents interact with the environment, that is, after the selection of pieces and cells, the environment delivers the global reward to the critic, which is the resultant of rewards/penalties. Then, the critic distributes the reward/punishment consequence among the agents based on the proposed method(s). Due to the increase in reward by the MAS at time $t^{new}$ relative to time $t^{old}$, this new distribution may be, in a way, that some inactive agents reach their TST values and therefore they are added to the active agents’ set, i.e., $MAS^{t}$. Consequently, at this stage, the set of active agents must be updated. Since, in our work, the Q-learning method was used to learn the agents, in the next step, the Q-table was updated for each agent. Given that each agent is responsible for completing a piece or several pieces of the puzzle based on their choice, in the next step, it should be checked which agent or agents completed their task(s). Therefore, in the next section, it will be examined whether $Ag_{i}$ (i∈{1,2,…,n}) has completed its task. If the task of the agent is completed, it will be removed from the list of active agents, i.e., it will not be employed in the next step. This concept is expressed in Equation (16):

(16) $${MAS}^{t+1}={MAS}^{t}-\left\{{Ag}_{j}:{Ag}_{j}\;\mathrm{Completes}\;\mathrm{its}\;\mathrm{task}\right\}$$

Of course, one may consider a situation in which this agent again participates in the problem-solving process. However, this may barricade other agents from participating in the problem-solving process. If $Ag_{i}$ has not completed its task, the agents are divided into two categories according to the new reward distribution. For the first category, the new distribution has not caused the received reward to exceed the TST. Consequently, the $SAg_{i}$ function has a false value for them and they have to wait for the new distribution of rewards again. On the other hand, for the agents of the second category, the $SAg_{i}$ function has a true value which means that they participate in the problem-solving process. After $MAS^{t+1}$ was calculated from Equation (16), it remains to see whether the puzzle was solved. If it was not solved, the above process is repeated with the new multi-agent system, $MAS^{t+1}$. This process will be repeated until the puzzle is solved and completed.

## 5. Evaluation and Results

The proposed methods are evaluated based on comparison to knowledge-based methods such as ranking, history-based, and dynamic methods. We developed C++ code in MATLAB R2021b, and PDToolbox was used to simulate the methods. The simulation was run on a 64-bit Windows 10 environment on a machine with an Intel Core i5-4200U Processor with a frequency of 2.3 GHz and 6 GB of RAM. The proposed methods were run in 100 episodes, and in each episode, based on the Q-learning, the iteration continued until it arrived at the target point. For this reason, Q-learning was used. Thus, in this process, the Q-table for each agent was updated, and the mentioned criteria were extracted.

To evaluate the proposed method, a 3 × 3 MsP was used. Obviously, the puzzle can be M × N. The number of pieces that exist in a puzzle section can be equal or non-equal. Here, we assumed that each section contained three pieces of the puzzle. Agents with different amounts of knowledge were used in the simulation. Each agent was responsible for putting three pieces of the puzzle in their place. We used the criteria outlined in [10,13] to evaluate our methods. It should be noted that, in the above references, these criteria were calculated for an agent operating in a multi-agent environment. In contrast, in this study, these criteria were considered as aggregate averages. The proposed methods were compared with the other methods according to the following criteria.

### 5.1. Group Learning Rate

The group learning rates’ average was considered as given in Equation (17):

(17) $${LR}^{t}=\left(\frac{1}{N}\right)\sum_{i=1}^{N}{LR}_{i}^{t}\;\;\;\;\;\;\;$$

Equation (18) denotes the individual learning rate of an agent:

(18) $${LR}_{i}^{t}=\frac{\left|Learnt(S_{i}^{t})\right|}{\left|S\right|}\;$$

The agent’s learning rate, as an individual, is defined as the rate of learner states given in Equation (19).

(19) $$Learnt\left(S_{i}^{t}\right)=$$

$$\left\{\forall a_{i}^{t}:feasible\left(a_{i}^{t},s_{i}^{t}\right)\to f_{a_{i}}^{\mathrm{suggested}}=f_{a_{i}}^{\mathrm{real}}\right\}$$

**Figure 3:**
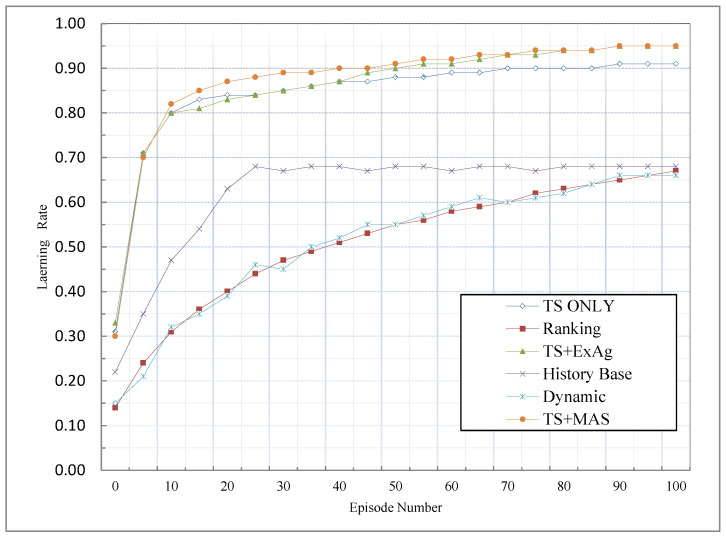
Comparison of MCA methods based on the group learning rate criterion.

In other words, $|Learnt(S_{i}^{t})|\;$ is the number of states that the agent will learn. Learning is defined as having the maximum value in the Q-Table in all states, in which case the correct action is selected through the greedy method. |S| shows the number of states available for each agent. This means that action $a_{i}^{t}$ is possible in-state $s_{i}^{t}$. Figure 3 shows the comparison results of the TS-only, TS + MAS, and TS + ExAg with other methods based on the learning rate criterion.

Since the agent with the highest knowledge for problem-solving is employed first, solving the section of the problem assigned to it gives a greater reward to the MAS for the total reward. Therefore, the group learning rate based on the proposed methods was much better than the other methods including the ranking method, history-based method, and dynamic method. Figure 3 confirms this issue. In the other methods, because agents reach their TSTs with a delay depending on the reward distribution, their learning, and consequently, the learning of the whole MAS, is delayed. Therefore, in Figure 3, we also see that the history base performs worse than the other start threshold-based methods and better than the ranking method and dynamic method. Therefore, it can be concluded that, in the proposed methods, the priority of awarding rewards to agents is based on their knowledge, so agents with higher knowledge will receive more rewards and will continue to perform the right action; and as a result, their learning will be higher. This causes agents with less knowledge (which reduces the learning rate) not to receive a reward, and as a result, they are excluded from the cycle of contributing to the work. These two issues cause the group learning rate to increase. Among the proposed methods, the ExAg method performs slightly better than the other proposed methods because it allocates the remaining reward to the knowledgeable agent. In addition, the TS + MAS method also performs slightly better than the TS-only method, which ignores the remaining reward because it allocates more rewards to agents with higher knowledge.

### 5.2. Confidence

The degree of confidence was the next criterion by which the suggested techniques were analyzed. The degree of confidence was acquired when completing the Q-table of the agent. Such a parameter was acquired by the subtraction of the second largest Q-table’s value from its maximum value. A bigger difference will result in a greater tendency of the agent to select the right actions.

If [$\;q\left(1\right)$, $q\left(2\right)$, $\;q\left(3\right)$,… , $q\left(|A|-1\right)$, $q\left(|A|\right)$] stand for the Q table values sorted in ascending order, then the agent’s confidence is calculated in accordance with Equation (20):

(20) $$\mathrm{Cnf}\left(S_{i}^{t}\right)=q\left(|A|\right)-q\left(|A|-1\right)$$

In the proposed methods, the more knowledgeable agents have greater priority over the other agents; therefore, compared to the proposed methods, they will receive a greater reward than the other agents. For this reason, less knowledgeable agents do not start to work; therefore, the agents will choose better. Since in the other methods less knowledgeable agents may start to work, more wrong choices happen, and this is the reason why the proposed methods perform better than the other methods. Furthermore, in the TS-only method, because the remaining reward is ignored after the agents reach their TST; therefore, more knowledgeable agents received lower rewards than in the TS + MAS and TS + ExAg methods. This is the reason why the confidence of the TS-only method was less than with the other two proposed methods.

**Figure 4:**
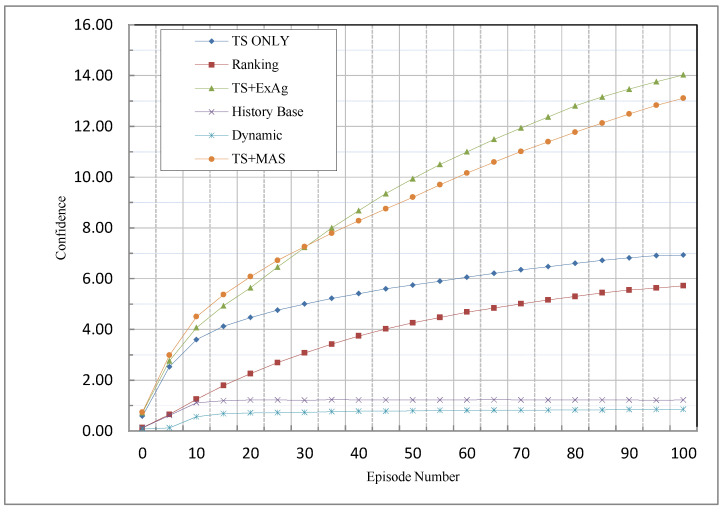
Comparison of MCA methods based on the confidence criterion.

Figure 4 illustrates the comparison between the proposed method and other methods based on the confidence criterion.

Figure 4 indicates that the ExAg method has the highest performance in this criterion. As mentioned earlier, this is because it assigns the remaining reward to the knowledgeable agent that has the best performance among the agents. The TS + MAS is basically similar to the TS + ExAg method, but it works slightly weaker than the ExAg method. However, its performance in this criterion is much better than the TS-only method. Among the existing methods, the best performance belongs to the ranking method. This is due to the fact that, in this method, agents with higher knowledge are given more attention. However, agents with less knowledge are also rewarded, which makes their performance much weaker than the proposed methods.

### 5.3. Expertness

The third criterion to compare the MCA-solving methods was expertness. Expertness refers to the difference between the number of times that an agent received a reward (selected the correct action) and the number of times that the same agent received a fine (selected the wrong action). Equation (21) expresses this criterion:

(21) $$Expertness=N_{r}\left(s_{i}^{t},a_{i}^{t}\right)-N_{p}\left(s_{i}^{t},a_{i}^{t}\right)$$

$N_{r}\left(s_{i}^{t},a_{i}^{t}\right)$: Number of times that the agent receives a reward.
$N_{p}\left(s_{i}^{t},a_{i}^{t}\right)$: Number of times that the agent receives a punishment.

**Figure 5:**
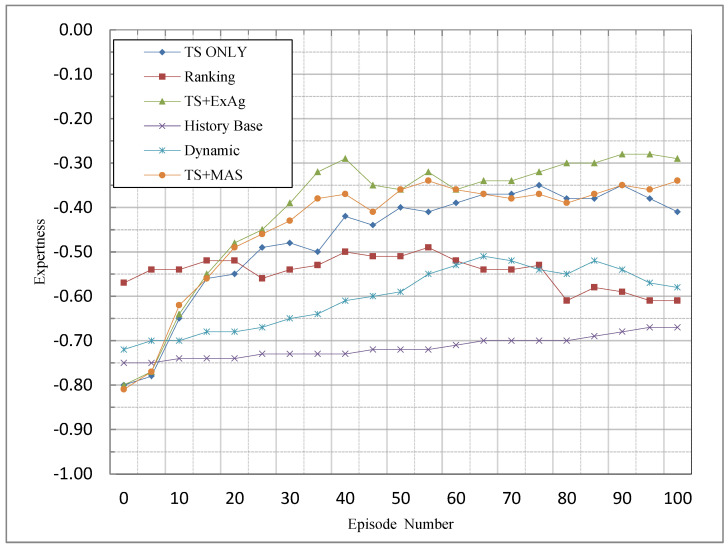
Comparison of MCA methods based on the expertness criterion.

Figure 5 illustrates the comparison between the proposed method and other methods based on the expertness criterion. The other methods give a reward to all agents (if possible), and they make no difference between them; therefore, the overall reward will be distributed among the agents. For this reason, the amount of the reward distributed among the agents will be reduced, so that the agents cannot reach their TST, and thus do not receive any rewards. In other words, they will be fined. Therefore, because there is no change in the allocation of rewards, the trend of the chart remains approximately constant. For the proposed methods, because the reward is distributed with respect to the TST, and consequently only the agent(s) with more knowledge receive the reward, in the first episodes, the MAS receives a low reward overall. Throughout the MAS activity, agents with higher knowledge gain more rewards, so in the reward distribution process, they cause the other agents to reach their TST, and consequently, the overall reward will be increased. For this reason, the frequency of the rewards will be increased while the blames will be decreased. Thus, in the diagram, the upward trend of the start threshold-based methods is evident in this criterion. Given that expertness serves on the basis of the number of punishments and rewards, and it does not serve on the basis of their amount, and because all the suggested techniques operate on the basis of the TST, the whole three suggested techniques outperform the other techniques.

From the point of view of this criterion, which can be seen in Figure 5, the TS + ExAg method has the highest performance. The reason for this is that the method allocates the remaining reward to the agent with the most knowledge. The performance of all three methods is weak in the initial episodes because the agents with high knowledge participate more in solving the problem, the number of times the reward is received increases over time, and in the final episodes, the performance is much better than the available methods in the literature. As the TS + MAS method attempts to assign the remaining reward to the agents who participate in solving the problem in proportion to their knowledge, it has a weaker performance than the TS + ExAg method, but its performance is better than the TS-only method, which ignores the remaining reward.

### 5.4. Certainty

Certainty was the fourth criterion employed in order to analyze the suggested techniques and other approaches. Certainty is estimated on the basis of Equation (22) and makes a comparison between the Q value in the action “*a*” and state “*s*” and other values of the “*s*” state on the basis of that

(22) $$\mathrm{Cert}\left(s_{i}^{t},a_{i}^{t}\right)=\frac{\exp\left(\frac{Q(s_{i}^{t},a_{i}^{t})}{T}\right)}{\sum_{a_{i}^{t}\in A}^{}\exp\left(\frac{Q(s_{i}^{t},a_{i}^{t})}{T}\right)}\;$$

Equation (23) specifies the *T* value for each episode.

(23) $$T=Max\left\{\frac{T_{0}}{1+\log\left(\mathrm{episode}\right)},T_{\min}\right\}\;$$

**Figure 6:**
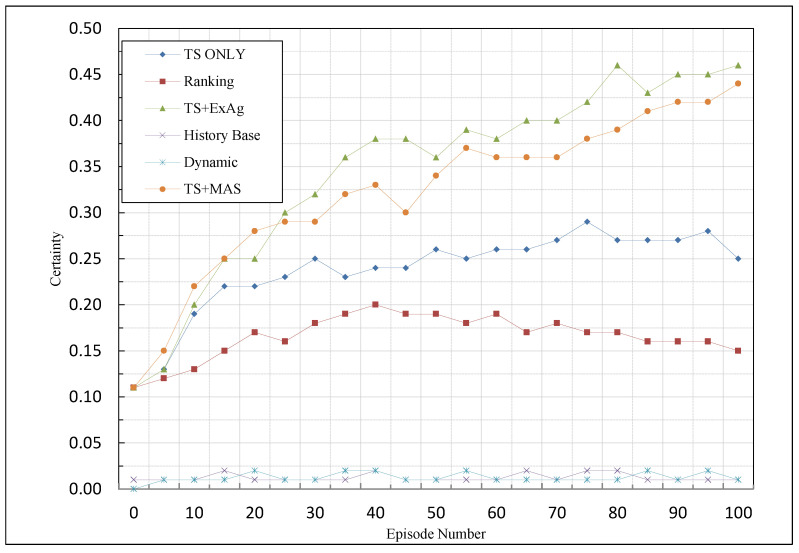
Comparison of MCA methods based on certainty criterion.

In accordance with [13], we set $T_{min}$ to 1 and $T_{0}$ to 10 in our experiments. The suggested techniques and the other approaches were compared in Figure 5 on the basis of the same criterion. Certainty acts on the basis of the values of the Q table, so it is evident that, if the values of this parameter will be fed later and have zero value in most episodes, the value of this parameter will be below. In the ranking technique (the best technique in certainty), given the TST constraint, the agents will reach the TST value and start operating in the environment with a delay; as a result, in this table, the number of zero values is high, while the number of non-zero values is lower in comparison with the other suggested techniques. For the same reason, on the basis of such a criterion, the other techniques performed poorly when compared to the suggested techniques. Among the proposed methods, because TS + MAS and TS + ExAg methods firstly assigned credits to agents based on the TST and redistributed the remaining rewards among the agents, if there was a surplus, they would have higher values in the Q table. For this reason, based on this criterion, those two methods worked better than the TS-only method, which only distributed the rewards until the TST was reached and the excess rewards were ignored. Figure 6 indicates the comparison of MCA methods based on the certainty criterion.

As shown in Figure 6, the TS + ExAg has the highest performance in this criterion. In the TS + ExAg method, the knowledgeable agent receives more rewards. Therefore, the best action will be taken with them, and this causes the values of the Q-table to increase. Since this criterion depends on the Q-table values, this criterion will also be increased. In the TS + MAS method, the remaining reward is distributed among the active agents, so the received reward by the knowledgeable agents is less than the received reward in the TS + ExAg method. For this reason, the performance of TS + MAS is weaker than the TS + ExAg; however, it acts better than the TS-only method, which ignores the remaining reward.

### 5.5. Efficiency

The fifth criterion employed to evaluate the techniques was the efficiency described by Equation (24). Efficiency refers to the number of times a positive reward is assigned to the agent. The credit assignment process, by the critic, should be conducted in a conservative manner to guide the agent towards the goal. This means that misleading the agents’ results in missing the target or spending more time to achieve the goal. Therefore, any non-zero assignment of the critic to the agent means that the critic accepts this risk and makes a judgment about the agent’s selective action.

(24) $$\mathrm{Eff}=\sum_{i=1}^{F}I\left(r_{i}^{t}\neq 0\right)\;\;\;\;\;\;\;$$

$$I\left(x\right)=\left\{\begin{array}{c} 1,\;\;\;x:\mathrm{True}\\ 0,\;\;\;x:\mathrm{False} \end{array}\right.\;$$

F:thenumberoffeedbacks

**Figure 7:**
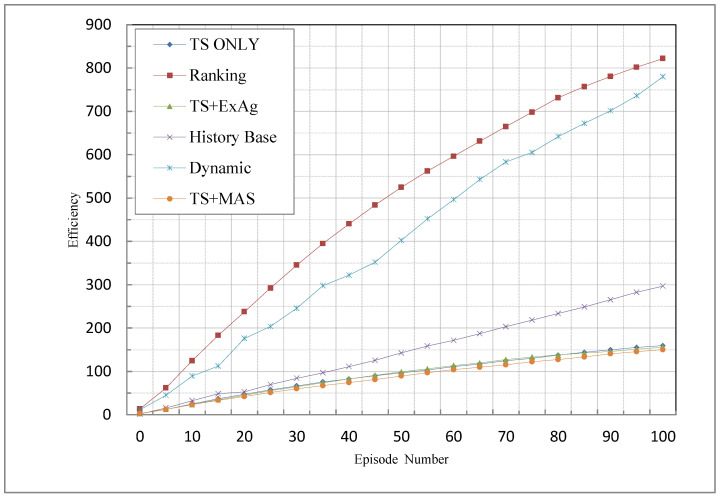
Comparison of MCA methods based on efficiency criterion.

The results of the comparison, according to this criterion, are shown in Figure 6. The ranking method performed very well because, in the ranking method (and two other methods), unlike the proposed methods, all agents were given a partial reward based on the overall reward rate. On the other hand, in the proposed methods based on the TST, the agents were rewarded only if their rewards were greater than the TST. As a result, the probability of receiving a positive reward was decreased, which led the ranking technique (the best technique among the other techniques) to outperform the threshold-based proposed techniques. In the proposed methods, as mentioned above, the implementation is based on a TST, so they are very similar to each other based on this criterion. In comparison with the proposed methods, as seen in Figure 7, their performances are very similar. This criterion is the only criterion in which the proposed methods have a weaker performance than the existing methods. This is because, according to Equation (24), efficiency is expressed in the form of the number of times that agents have received non-zero rewards. In the proposed methods, agents with low knowledge were not rewarded, so in this criterion, the proposed methods have a weaker performance.

### 5.6. Correctness

The last criterion for making comparisons between the methods was correctness. Correctness can be calculated based on different interpretations. A more flexible definition of the correctness of an assignment is achieved by a threshold. In that case, if the difference between the completed assignment and the actual assignment is less than this threshold, then it is counted as a correct assignment; otherwise, it is considered to be a mistake. Another definition for the correctness of choice is based on the sign only (not the magnitude). If the signs of the achieved assignment and the actual assignment are the same, then the assignment is correct. In this study, we used the threshold-based definition. Equation (25) illustrates this matter:

(25) $$\mathrm{Corr}=\sum_{i=1}^{F}I(|r_{i}^{t}-r_{i\;\mathrm{cor}}^{t}|<T)\;$$

$$I\left(x\right)=\left\{\begin{array}{c} 1,\;\;\;x:\mathrm{True}\\ 0,\;\;\;x:\mathrm{False} \end{array}\right.\;$$

$$r_{i}^{t}:\;\mathrm{The}\;\mathrm{done}\;\mathrm{assignment}$$

$$r_{i\;\mathrm{cor}}^{t}\;:\;\mathrm{The}\;\mathrm{actual}\;\mathrm{assignment}$$

F:TheNumberofFeedbacks

T:ThresholdValue

**Figure 8:**
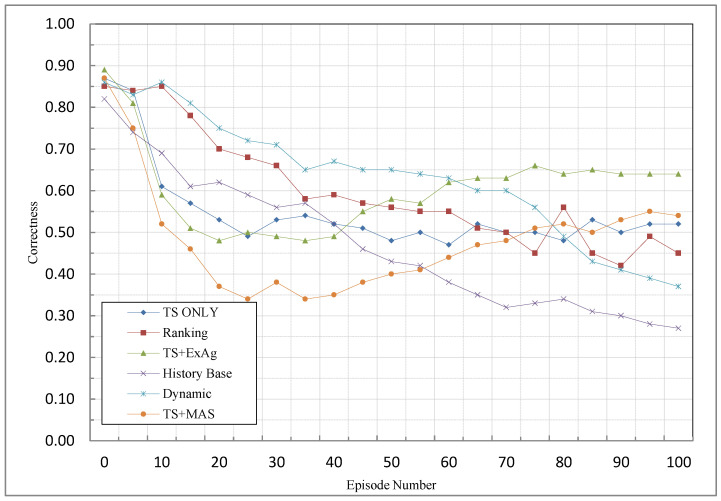
Comparison of MCA methods based on correctness criterion.

Figure 8 indicates the comparison of the proposed methods and other methods based on this criterion. In this diagram, given the nature of the proposed methods for problem-solving, which first focuses on more knowledgeable agents, and then less knowledgeable agents are used, even if the correct action is performed by the less knowledgeable agent, most of the rewards will be assigned to the agent(s) with higher knowledge. Therefore, as expected, the correctness in this chart will initially go downhill; this is because the probability of the reward being assigned to less knowledgeable agents which ae correctly performing their task is low in the first episodes. In continuation of the diagram and higher episodes, because gaining rewards is increased, the rewards assigned to these types of agents (less knowledgeable agents) are also increased too. For this reason, in higher episodes, the diagram tends to increase. Here, given the distribution of rewards among the agents, and because in the other methods, it is possible to credit assignment to most agents, they work better in the first episodes. However, since there is less chance of earning more rewards for the agents in this method, the other methods in the higher episodes are downward, and the proposed methods perform better than these methods. Between the proposed methods, the TS + ExAg method also performs better due to the assignment of a higher reward to the agent with higher knowledge. Therefore, this results in gaining higher rewards and better distribution among the agents which increases the likelihood of the reward being assigned to less knowledgeable agents that have correctly performed their tasks, which in turn results in improved correctness.

As can be seen in Figure 8, the Ts + ExAg has the highest performance among all methods. Because this criterion depends on the time that the reward is assigned correctly so, in the first episodes, the performance of the proposed methods is down. After that, and especially in the TS + ExAg method, because knowledgeable agents are used, the performance of the proposed methods is improved. Because of this, in the TS + MAS method, the remaining reward is assigned to active agents, so it is possible that the agents that have a low effect on MASs’ success receive the reward. Therefore, the performance of this method, according to correctness criteria, is lower than the TS + ExAg method. However, this method works better than the TS-only method.

Table 2 summarizes the comparison of the proposed methods based on the criteria discussed in the evaluation section. In addition, it shows the advantages and disadvantages of any method.

## 6. Discussion

Many systems can be mapped into the MAS, and based on this mapping, one can use MAS capabilities to solve the system’s problem. One of these systems is smart cities. One of the important problems in smart cities is resource allocation. Although many solutions have been developed to solve this problem, resource allocation is still a critical problem in smart cities that can be extended.

On the other hand, there are many smart devices (consumers) in smart cities with different knowledge from that of the system and intelligence level. Generally, the resources in smart cities are limited, and they are usually less than what the consumers need. Therefore, in order to optimize the use of resources, we should look for efficient methods to use these available resources. In traditional methods that were proposed to solve the resource allocation in smart cities, it is attempted that the resources are assigned to all consumers, and the system’s performance has been paid less attention. Therefore, one aspect of resource allocation in smart cities is performance improvement, which needs to be further investigated. To this end, we proposed our methods in this paper.

We mapped the smart city into the MAS. Due to the fact that the resources (global reward) should be assigned to the consumers of the system as agents, the resource allocation is turned into the MCA problem. In addition, as the resources are less than the consumers’ request, we used the bankruptcy concept to solve the resource allocation problem. In this paper, based on the bankruptcy concept, we presented an algorithm called “RevAP” to solve the MCA problem. Based on this algorithm, and three methods of TS-only, TS + MAS, and TS + ExAg were developed to solve the MCA.

In order to evaluate these methods, six criteria, namely the group learning ratio, confidence, expertness, certainty, efficiency, and correctness, were used to evaluate these methods. As these methods are based on knowledge, we compare them with the knowledge-based methods such as the ranking, history-based, and dynamic methods. In the existing methods, the global reward was assigned to all agents, and the agents with less knowledge may receive part of the reward. Receiving rewards from agents with less knowledge leads the MAS to weak performance. Therefore, in order to overcome this issue, we applied the proposed methods. In the proposed methods, the knowledgeable agents receive more rewards than the fewer knowledge agents, and fewer knowledge agents may not receive any reward or less reward. Therefore, the performance of the MAS will be increased.

The simulation results indicate that the MAS performance in five parameters, i.e., group learning ratio, confidence, expertness, certainty, and correctness, is improved significantly. The results of the simulation also show that, among the proposed methods, the TS + ExAg method has the highest performance. This is because, in this method, the remaining reward is assigned to the knowledgeable agent or agents, which causes the performance of the agent or agents in question to improve, and as a result, the performance of the MAS is improved. Therefore, it can be concluded that the proposed methods are suitable for smart systems such as smart cities in which there are many smart and intelligent devices (as consumers) with different intelligence levels. In addition, unlike the traditional methods for resource allocation in smart cities, our methods are focused on increasing the system’s performance.

## 7. Conclusions and Technical Outlook

### 7.1. Conclusions

Smart cities are among the symbols of the future world that contains many subfields such as smart transportation, smart healthcare, smart homes, smart factory, etc. In almost all of these subfields, resource allocation is an essential issue. On the other hand, an MAS is a suitable tool for modeling intelligent and distributed systems such as smart cities. As such, in this paper, smart cities or their subfields are considered MASs. In addition, resource allocation in smart cities is considered as MCA which is a part of the MARL process. In this paper, to solve the MCA by considering the TST and MSP, three methods, namely TS-only, TS + MAS, TS + ExAg, were proposed based on the bankruptcy concept. In addition, to consider a more realistic scenario, the idea of MSP was exploited for the case of a non-uniform environment and problem, and a TST constraint was introduced which causes the agents to start working from a non-zero point. To evaluate these methods, six criteria, i.e., group learning ratio, confidence, expertness, certainty, efficiency, and correctness were used and calculated for MASs. In comparing these three methods with the other methods based on the learning rate, the TS + MAS method worked a little better than the other two. However, because we used the bankruptcy concept in these methods, they performed better than the history-based method (as the best method among the other methods in learning rate) by 40 percent in the learning rate. Considering the confidence criteria, TS + MAS and TS + ExAg outperformed TS-only; nevertheless, all of them are still superior to the other methods; for example, the TS + ExAg performed approximately five times better than the ranking method. Based on the expertness criterion, all three proposed methods were better than the other methods. The simulation results showed that TS + MAS and TS + ExAg performed better than TS-only in terms of the certainty criterion, but again all of them performed better than the ranking method as the best method in the other methods. Efficiency was the only criterion based on which the other methods showed better performance than the suggested methods, which was because this criterion is dependent on the number of rewards received by the agents. The last evaluation criterion was correctness, based on which the other methods experienced a better performance at first. Nonetheless, over time and in higher episodes, the suggested methods outweighed the other methods, and among them, TS + ExAg was the most superior to the other methods. As a further work, it is suggested that the agents are categorized and a critic is considered for each category. Then, a suitable method of bankruptcy is used for each category according to its conditions. Furthermore, in this case, the critics can be considered hierarchically and this categorization idea can be applied to them so that the set of critics itself can be considered as an MAS. In this study, six parameters were used to evaluate and compare the methods.

### 7.2. Technical Outlook

The use of MASs for modeling systems and the real world is increasing, an evident example of which is that of smart cities. Many MASs can be considered in smart cities where RL, by distributing rewards among them, plays an important role. Examples of this type of system include smart transportation, smart healthcare, smart homes, and smart factories, which are subfields of smart cities. Improving such systems is one of the most important goals of their developers. The methods proposed in this paper can be used to achieve this goal. If a system can be considered an MAS and there is a resource allocation problem in this system, then the outlined methods can be readily applied. Examples of this type are abundant in a variety of fields. Among the situations in which the proposed methods can be used, there is smart cities resource allocation. Smart cities can be considered MASs in which each consumer is an agent, and the resource is a global reward that should be distributed between the agents. The results indicate that applying the proposed method increases the efficiency of the system.

## Figures and Tables

**Table 1:** Comparing the MCA solving methods.

Method	Equal Approach	Fair Approach	Knowledge-Based	Efficiency Performance	Contribution	Advantage	Disadvantage	Best Performance
Ranking	×	×	✓	Partly	Introducing the parameters to improve the knowledge-based method to solve the MCA	The agents’ knowledge is used	The possibility of assigning a reward to the less knowledgeable agents	Performance matters somewhat
History base	×	Partly	✓	×	Introducing a model-based method to solve the MCA	Modeling the MCA by a graph	Weakness in scalability	Model-based
Dynamic	×	×	✓	Partly	Introducing a new parameter to solve the MCA	The agents’ knowledge is used	Low accuracy	The numbers of features are low
TS-only	×	×	✓	✓	1. Introducing the TST constraint 2. Introducing the MsP problem 3. Introducing the bankruptcy method to solve the MCA problem	Improves the system’s performance	Ignores the remaining reward	The performance is important
TS + MAS	×	×	✓	✓	1. Introducing the TST constraint 2. Introducing the MsP problem 3. Introducing the bankruptcy method to solve the MCA problem	Improves the system’s performance	Training phase is needed	The performance is important
TS + ExAg	×	×	✓	✓	1. Introducing the TST constraint 2. Introducing the MsP problem 3. Introducing the bankruptcy method to solve the MCA problem	Improves the system’s performance	Training phase is needed	The performance is important
LeCTR	✓	×	×	×	Peer-to-peer teaching in cooperative MARL	Simple	Unfair and inefficient	Simplicity is important
IS	✓	×	×	×	Enhance the coordination among the agents	Simple	Unfair and inefficient	Simplicity is important
MAK-TD	×	✓	×	×	A coupled gradient descent is adopted for developing a method to approximate the reward	The possibility of using heterogeneous agents	Potential information loss in high dimensions	Fairness is important
SQDDPG	×	✓	×	×	Applies the “Shapley” method to solve the MCA problem	Global reward distribution is guaranteed	High complexity	Fairness is important

**Table 2:** Comparing the proposed methods to solve the MCA.

Proposed Method	Remaining Reward	Advantage	Disadvantage	Learning Rate	Confidence	Expertness	Certainty	Efficiency	Correctness
TS-only	Ignored	Simpler than TS + MAS and TS + ExAg	Ignores the remaining reward	Approximately similar to the other proposed methods	Third best	Third best	Third best	Approximately similar to the other proposed methods	Third best
TS + MAS	Assigns to active agents based on their knowledge	Fairer than TS-MAS and TS + ExAg	More complicated than the other proposed methods	Similar to the other proposed methods	Second best	Second best	Second best	Similar to the other proposed methods	Second best
TS + ExAg	Assigns to the knowledgeable agent	The best performance between the proposed methods	High dependence on the knowledgeable agents	Similar to the other proposed methods	The best	The best	The best	Similar to the other proposed methods	The best

## Data Availability

Not applicable.
